# Obesity accelerates age defects in B cells, and weight loss improves B cell function

**DOI:** 10.1186/s12979-023-00361-9

**Published:** 2023-07-17

**Authors:** Daniela Frasca, Maria Romero, Alain Diaz, Bonnie B. Blomberg

**Affiliations:** 1 Department of Microbiology and Immunology, University of Miami Miller School of Medicine, RMSB 3153, 1600 NW 10thAve, Miami, FL 33136 USA; 2 Sylvester Comprehensive Cancer Center, University of Miami Miller School of Medicine, Miami, FL USA

**Keywords:** Aging, Obesity, B cells, Humoral immunity, Influenza vaccination

## Abstract

**Background:**

We have previously shown that obesity accelerates age-associated defects in B cell function and antibody production leading to decreased secretion of protective antibodies and increased autoimmunity. We wanted to evaluate if obese adults enrolled in a voluntary weight reduction program had higher protective and lower autoimmune antibody responses similar to those observed in lean adults.

**Methods:**

Experiments were performed using blood isolated from an established cohort of female lean adult and elderly individuals, as well as from the blood of female adults with obesity, before and after a voluntary weight reduction program in which their Body Mass Index (BMI) was reduced 10–34% in 12 months. All participants were vaccinated with the Trivalent Inactivated Influenza vaccine. Serum samples were evaluated for the presence of pro-inflammatory cytokines and adipokines, vaccine-specific antibodies and autoimmune antibodies. We evaluated the composition of the B cell pool by flow cytometry, the expression of RNA for class switch transcription factors and pro-inflammatory markers by qPCR, the in vitro secretion of pro- and anti-inflammatory cytokines and their capacity to induce pro-inflammatory T cells.

**Results:**

Obesity, similar to aging, induced increased serum levels of pro-inflammatory cytokines and autoimmune antibodies, while vaccine-specific antibodies were reduced. In agreement with the serum results, the B cell pool of obese adults and elderly individuals was enriched in pro-inflammatory B cell subsets and was characterized by higher expression of markers associated with cell senescence, higher levels of T-bet, the transcription factor for autoimmune antibodies and lower levels of E47, the transcription factor associated with protective responses to the influenza vaccine. B cells from obese adults and elderly individuals were also able to secrete inflammatory cytokines and support the generation of inflammatory T cells. All these pro-inflammatory characteristics of B cells from obese individuals were significantly attenuated, but not completely reversed, by weight loss.

**Conclusions:**

Although the results from our small observational study show that obesity-induced dysfunctional B cell responses, similar to those occurring during aging, are ameliorated in some but not all obese individuals after weight loss, the effects of body weight loss on mechanistic pathways are largely missing and deserve further investigation.

## Background

Our previously published findings have indicated that obesity accelerates age-associated defects in protective antibody responses and induces autoimmunity [1–4]. Aging is also associated with dysfunctional humoral immunity that leads to compromised antibody responses to infectious agents and vaccines, and increased autoimmunity [5]. The mechanisms through which obesity and aging affect humoral immunity appear to be in large part overlapping, as seen in both cases for combined defects in B cells, T cells and other immune cells. The obesity- and aging-driven defects observed in immune cells are linked to the increased chronic low-grade systemic inflammation, known as inflammaging [6], that induces intrinsic inflammation in immune cells and is therefore negatively associated with a functional immune system, healthspan and longevity in both mice and humans [7]. Both obesity and aging are significant risk factors for conditions and diseases typical of old age, such as type-2 diabetes mellitus, cancer, cardiovascular disease, atherosclerosis, and autoimmune diseases [8], further confirming that they may share cellular and molecular pathways and underlying mechanisms [9].

When we evaluated the effects of obesity on the serum antibody response to the influenza vaccine in young and elderly healthy individuals, we found a negative correlation between Body Mass Index (BMI) and serum specific antibodies [2, 3]. Interestingly, we found that the vaccine-specific response of young obese individuals was not different from that of elderly lean individuals. These results suggested to us for the first time that obesity induces age defects in the humoral response to the influenza vaccine, making obesity a biomarker of accelerated aging at least for antibody responses. In particular, we found that obesity, similar to aging, induced defects in class switch recombination, the process necessary for the generation of high affinity secondary antibodies [10], due to reduced activation-induced cytidine deaminase (AID), the enzyme of class switch recombination and somatic hypermutation, and E47, a key transcription factor that regulates AID [11]. Both AID and E47 are decreased in B cells isolated from the blood of obese young and elderly individuals as compared to lean and young controls, respectively [3]. Also for these measures, the response of elderly lean and young obese individuals was similar. In further support of our hypothesis, we have also found comparable amounts of IgG antibodies with autoimmune specificity in serum samples of young obese and elderly lean individuals [4].

Although public health campaigns have advocated the importance of weight loss to improve protection from infection and increase the effects of vaccination, there is still no strong evidence demonstrating that weight loss leads to a stronger humoral immune response to infection and vaccination. Therefore, we aimed to investigate the impact of a voluntary weight reduction program, in which the BMI of obese adults was reduced 10–34% in 12 months, on humoral immunity. Previously published results in overweight/obese human beings have shown that caloric restriction and weight loss over a time of 6–24 months reduce inflammaging [12–14]; improve thymic function [14]; increase T cell proliferation and delayed-type hypersensitivity [15]. However, to our knowledge, results on the effects of weight loss on B cell function have not been published yet. Our results, obtained on a small cohort of female individuals with obesity, are promising and show that weight loss may have a significant impact on the improvement of the quality of humoral immunity.

## Results and discussion

### Obesity and aging similarly increase serum levels of pro-inflammatory cytokines and adipokines, which are then decreased, at least in part, by weight loss

**Fig. 1:**
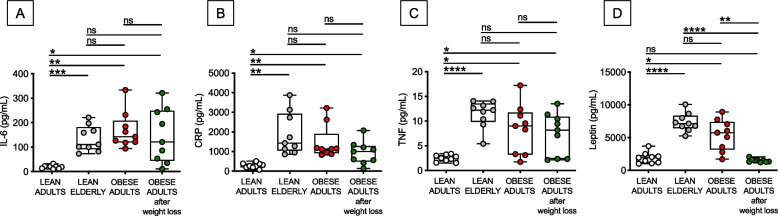
Weight loss decreases the serum levels of pro-inflammatory cytokines and adipokines. IL-6 (**A**), CRP (**B**), TNF (**C**) and leptin (**D**) were detected in serum samples of the 4 groups of participants by ELISA. Mean comparisons between groups were performed by two-way ANOVA: *p < 0.05, **p < 0.01, ***p < 0.001, ****p < 0.0001

We have previously shown that obesity accelerates age-associated defects in humoral immunity and B cell function [2, 3]. This occurs through increased systemic inflammation, inflammaging, and B cell intrinsic inflammation, making B cells from obese individuals similar to those from elderly individuals. Some published observations have shown that in adults that are owerweight or moderately obese, serum levels of multiple inflammatory markers were decreased after a mild weight reduction of about 5–10% [16, 17]. Therefore, we first evaluated if weight reduction was associated with reduced levels of serum pro-inflammatory cytokines in our cohort of obese individuals. We measured serum levels of IL-6, CRP, TNF-α and leptin in obese adults before and after weight loss and compared these values to those from lean adults and elderly individuals. Results in Fig. 1 show that these inflammatory markers were significantly and similarly increased by aging and obesity, further confirming our previously published observations. Although weight loss was effective in reducing in some individuals serum levels of IL-6 (A), CRP (B) and TNF (C), these values were still significantly different from those in lean adults. The only exception was leptin (D), increased by aging and obesity, and decreased after weight loss to the levels observed in lean adults. These results provide evidence of the benefits of weight loss on the decrease of serum levels of inflammatory markers in some but not all individuals and suggest that longer time on diet may be needed in those individuals who are still showing effects of obesity on serum inflammatory markers.

### Obesity, similar to aging, induces dysfunctional antibody responses which can be reversed, at lest in part, by weight loss

**Fig. 2:**
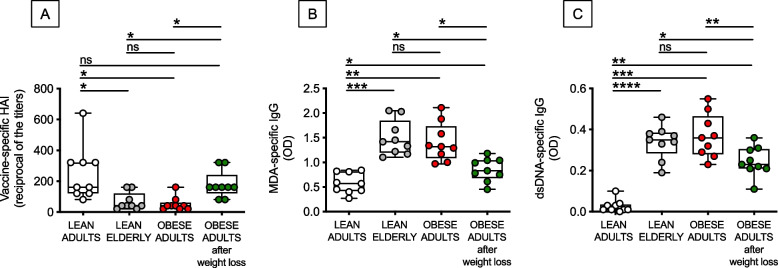
Weight loss is associated with increased serum levels of protective responses to the influenza vaccine and reduced autoimmune antibodies. Serum samples were collected from the 4 groups of individuals. Vaccine-specific antibodies were evaluated by HAI (**A**). Results show the reciprocal of the titers 4 weeks after vaccination.MDA-specific (**B**) and dsDNA-specific (**C**) autoimmune antibodies were measured by ELISA. Mean comparisons between groups were performed by two-way ANOVA: *p < 0.05, **p < 0.01, ***p < 0.001, ****p < 0.0001

Then, we tested serum antibody responses of obese adults before and after weight loss and compared these responses to those observed in lean individuals of different ages. Serum samples were evaluated for the presence of protective antibodies after influenza vaccination and of pathogenic autoimmune antibodies. We measured vaccine-specific antibodies by HemAgglutination Inhibition (HAI) assay which is the best correlate for vaccine protection. On the same serum samples we also measured by ELISA the presence of autoimmune antibodies specific for malondehyldehyde (MDA) and for double strand (ds)DNA, two autoantigen specificities associated with obesity and obesity-driven increased oxidative stress and lipid peroxidation (measured by MDA) [18, 19] and increased DNA damage (measured by dsDNA) [20], leading to the release of antibodies of these specificities in blood. Results in Fig. 2 show that vaccine-specific antibodies were decreased by both aging and obesity. Weight loss enhanced antibody levels to those observed in lean adults (A). As to autoimmune antibodies, both MDA-specific (B) and dsDNA-specific (C) antibodies were significantly increased by aging and obesity. Weight loss decreased these antibodies although levels were still different from those of lean adults.

Importantly, vaccine-specific antibodies were positively correlated with percent of weight loss (*r* = 0.76, *p* = 0.02), whereas autoimmune antibodies were negatively correlated with percent of weight loss, with a trend shown for MDA-specific antibodies (*r* = -0.49, *p* = 0.05) and a significant correlation shown for dsDNA-specific antibodies (*r* = -0.80, *p* = 0.01) (data not shown). These results altogether confirm the benefits of weight loss on systemic humoral immunity.

### Obesity and aging induce similar changes in the expression of transcription factors for class switch and these changes can be reversed, at least in part, by weight loss

**Fig. 3:**
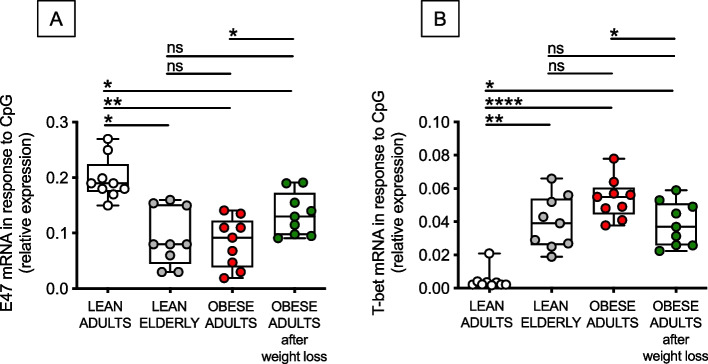
Obesity and aging induce similar changes in the expression of transcription factors for class switch and these changes can be reversed, at least in part, by weight loss. B cells were isolated from PBMCs using CD19 microbeads and positive selection. B cells (10^6^ cells/ml) were cultured with CpG for 1 day. The mRNA was extracted and qPCR performed to evaluate expression of E47 (**A**) and T-bet (**B**) mRNA, respectively. Results show qPCR values which are measures of RNA expression of target genes, relative to the housekeeping gene GAPDH, calculated as 2^−ΔCts^. Mean comparisons between groups were performed by two-way ANOVA: *p < 0.05, **p < 0.01, ****p < 0.0001

Next, we isolated B cells from the peripheral blood and evaluated the expression of transcription factors for class switch and antibody secretion after stimulation with CpG. We measured by qPCR mRNA levels of E47, associated with protective responses to infections and vaccines, and T-bet, associated with autoantibody secretion. B cell cultures were harvested at day 1 of stimulation, a time point that we found optimal in our previously published work [3, 21, 22]. Based on results in Fig. 2, we hypothesized not only that the age- and obesity-driven decreases in the serum influenza vaccine response were associated with decreased expression of E47, with a concomitant increase in T-bet (responsible for the increase of serum autoimmune antibodies), but also that weight loss induced a change in the transcriptional profile of B cells from obese adults improving E47 and decreasing T-bet. Results in Fig. 3 confirmed our hypothesis showing a decreased expression of E47 mRNA, and an increased expression of T-bet mRNA, in CpG-stimulated B cells from elderly and obese individuals as compared to lean adult controls. Moreover, our results showed the effects of weight loss on the increase of E47 mRNA expression (A), and on the decrease in T-bet mRNA expression (B) in some but not all individuals.

These results altogether show the benefits of weight loss on mRNA expression of transcription factors involved in class switch, although their expression in B cells from obese individuals undergoing weight loss is still different from that observed in B cells from lean adults, suggesting again that longer time on diet may have better effects.

### Obesity, similar to aging, increases blood frequencies of inflammatory Double Negative (DN) B cells, associated with reduced responses to the influenza vaccine and with increased autoimmunity, which are then decreased by weight loss

The composition of the peripheral B cell pool influences the profile of serum antibodies of an individual, and it has been shown that serum vaccine-specific antibodies are reduced in lean elderly [23–27] and in obese adults [3] as compared to lean adults, in part due to a redistribution of B cell subsets in the circulating B cell pool. This redistribution leads to the expansion with aging and obesity of the subset of the most inflammatory B cell subset, called Double Negative (DN) B cells, which are found increased in the blood of healthy elderly individuals [28–30], individuals with obesity [1–3], with autoimmune diseases [31–37], and with both chronic [38–40] and acute [41] infectious diseases. We have previously demonstrated that the expansion of this subset is linked to the increased secretion of autoimmune antibodies [1, 2].

**Fig. 4:**
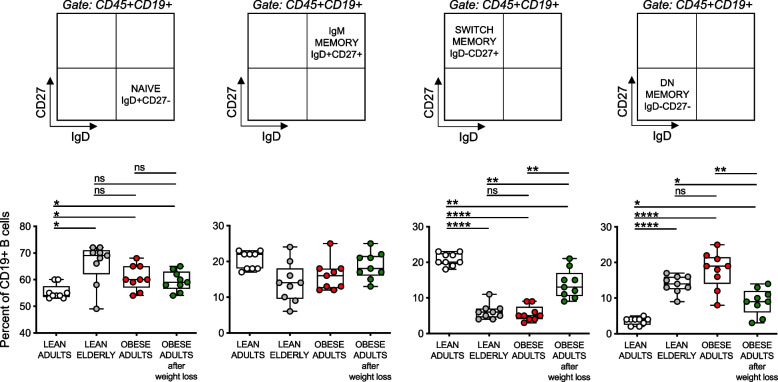
Obesity, similar to aging, increases blood frequencies of inflammatory DN B cells, associated with reduced responses to the influenza vaccine and with increased autoimmunity, which are then decreased by weight loss. Unstimulated PBMCs were membrane stained with Live/Dead detection kit and anti-CD45/CD19/CD27/IgD antibodies. Top. Gating strategies to evaluate the major B cell subsets. Bottom. Frequencies of naïve, IgM memory, switched memory and DN B cells from all donors (means ± SE), gated on live CD45 + CD19 + , are shown. Mean comparisons between groups were performed by two-way ANOVA: *p < 0.05, **p < 0.01, ****p < 0.0001

We therefore measured the frequencies of the major B cell subsets present in the blood of the 4 groups of individuals. Our results in Fig. 4 confirm these previously published results. Figure 4 top shows gating strategies to evaluate the different B cell subsets after staining B cells with anti-CD19, anti-CD27 and anti-IgD antibodies to evaluate the frequencies of the major B cell subsets. Results from all the participants are shown in Fig. 4 bottom. Briefly, naïve B cells are significantly increased, while switched memory B cells are significantly decreased, by aging and obesity, in agreement with the age- and obesity-driven decrease in class switch. Conversely, DN B cells are increased by aging and obesity, as expected due to the increased autoimmune antibody secretion with age and obesity. No changes on IgM memory B cells were observed. The frequencies of total B cells were decreased by aging but not by obesity (not shown). Weight loss induced increased frequencies of switch memory and decreased frequencies of DN B cells as compared to obese controls, although the frequencies of these B cell subsets were still significantly different from those in lean adults. No weight loss-driven differences in the frequencies of total B cells were reported.

These are to our knowledge the first results showing a redistribution of B cell subsets with increased frequencies of switched memory B cells and decreased frequencies of DN B cells in the blood of obese individuals undergoing weight reduction, as compared to obese controls.

### Weight loss is also associated with decreased expression of markers of the senescence-associated secretory phenotype, SASP, in unstimulated B cells

**Fig. 5:**
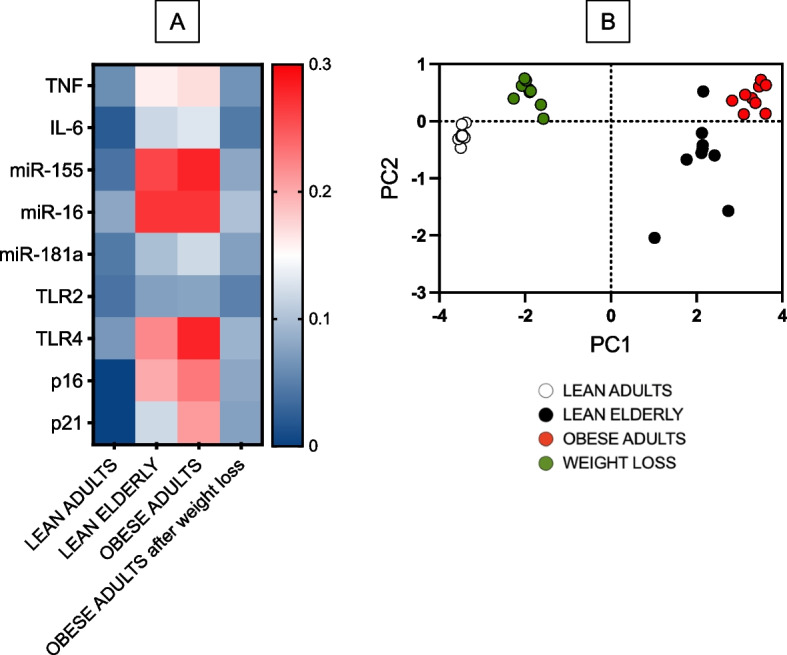
Weight loss is associated with decreased expression of markers of the senescence-associated secretory phenotype, SASP, in unstimulated B cells. B cells were isolated from PBMCs using CD19 microbeads and positive selection. B cells, left unstimulated, were resuspended in TRIzol, then the RNA was extracted and the expression of SASP markers detected by qPCR to evaluate expression of RNA for pro-inflammatory cytokines, pro-inflammatory miRs, TLRs and cell cycle regulators p16^INK4^, p21^CIP1/WAF1^. **A**. qPCR values are measures of RNA expression of target genes, relative to the housekeeping genes GAPDH or U6 (for miRs quantification), calculated as 2^−ΔCts^. **B**. PCA analysis showing the variation among groups explained by PC1 and PC2. Each symbol indicates an individual

Because DN B cells are characterized by high RNA expression of several pro-inflammatory markers that are markers of the SASP, contributing significantly to inflammaging, we measured levels of transcripts of pro-inflammatory cytokines (TNF, IL-6), micro-RNAs, miRs (miR-155, miR-16, miR-181a), Toll-like Receptors (TLR2 and TLR4) and cell cycle inhibitors and markers of proliferation arrest (p16^INK4^, p21^CIP1/WAF1^) [29, 42]. Results in Fig. 5A show that the levels of RNA expression of all the pro-inflammatory markers are higher in B cells from both obese adults and lean elderly as compared to B cells from lean adults, with weight loss being able to reduce these levels as compared to B cells from obese adults, although levels were never comparable to those observed in B cells from lean adults. In the PCA analysis in Fig. 5B distinct clustering of SASP markers in B cells from the 4 groups of individuals are shown, clearly indicating a beneficial effect of weight loss.

These results showing the effects of weight loss in decreasing the expression of SASP markers in unstimulated B cells are of significant relevance because the members of the SASP have systemic effects as they are released in the circulation and sustain and propagate systemic inflammation. Moreover, expression of SASP markers in unstimulated immune cell is negatively associated with the capacity of the same cells to be optimally stimulated either in vivo or in vitro, as we [43] and others [44, 45] have previously demonstrated for B cells and T cells, respectively.

### Obesity, similar to aging, increases in vitro secretion of the pro-inflammatory cytokine IL-6 and decreases that of the anti-inflammatory IL-10, and weight loss ameliorates but does not completely reverse this secretory phenotype

**Fig. 6:**
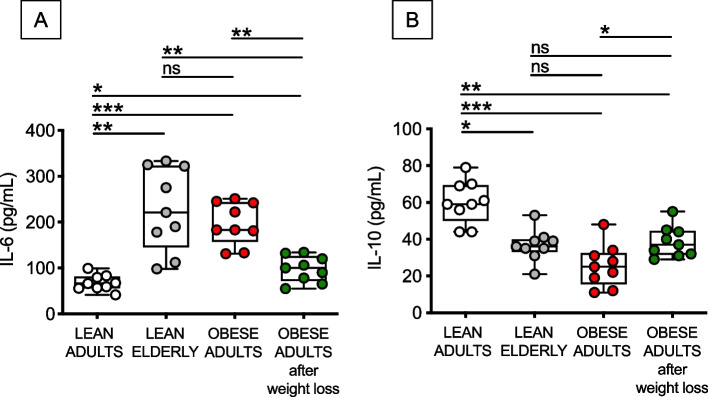
Obesity, similar to aging, increases in vitro secretion of the pro-inflammatory cytokine IL-6 and decreases that of the anti-inflammatory IL-10, and weight loss ameliorates but does not completely reverse this secretory phenotype. B cells were isolated from PBMCs using CD19 microbeads and positive selection. B cells (10^6^ cells/ml) were cultured with CpG for 2 days. IL-6 (**A**) and IL-10 (**B**) were measured in culture supernatants by ELISA. Mean comparisons between groups were performed by two-way ANOVA: *p < 0.05, **p < 0.01, ***p < 0.001

Because of the increased expression of the frequencies of DN B cells, as well as of the increased RNA expression of SASP markers, in B cells of lean elderly and obese adults as compared to B cells from lean adults, we evaluated the effects of aging and obesity, and of weight loss, on the in vitro secretion of pro- and anti-inflammatory cytokines. Briefly, B cells isolated from the blood of the 4 groups of individuals were stimulated with CpG for 48 h, then supernatants collected and evaluated for the presence of IL-6 or IL-10 by ELISA. Results in Fig. 6 show that IL-6 is increased (A), while IL-10 is decreased (B), in supernatants of B cells from lean elderly and obese adult individuals as compared to B cells from lean controls. Weight loss was able to significantly decrease IL-6 and significantly increase IL-10, but not to the levels observed in B cells from lean controls. Nevertheless, our results indicate a significant reduction of the intrinsic inflammatory profile of B cells from obese adults after weight loss which suggests a better capacity to improve protective, while reducing pathogenic humoral immunity.

### B cells from obese adults and lean elderly individuals, but not from lean adults, support T cell inflammation and this effect is reduced by weight loss

**Fig. 7:**
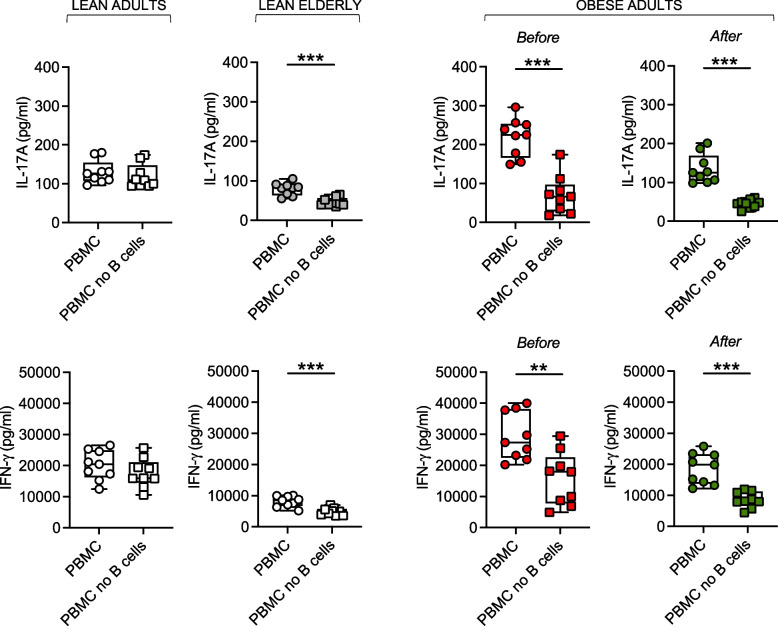
B cells from obese adults and lean elderly individuals, but not from lean adults, support T cell inflammation and this effect is reduced by weight loss. PBMCs were stimulated for 48 h with plate-bound anti-CD3 in the presence or absence of B cells, which were removed by magnetic sorting using CD19 microbeads (< 0.1% B cells in PBMC after selection). IL-17A (top) and IFN-γ (bottom) were measured in culture supernatants by ELISA. Mean comparisons between groups were performed by Student’s t test (two-tailed). **p < 0.01, ***p < 0.001, ****p < 0.0001

We finally evaluated the capacity of B cells from obese adults, before and after weight loss, to support T cell inflammation, as compared to B cells from lean individuals of both age groups. Briefly, PBMC with or without B cells were stimulated for 48 h with plate-bound anti-CD3 antibodies, then secretion of IL-17A and IFN-γ was measured by ELISA in culture supernatants. Results in Fig. 7 show that the removal of B cells from PBMC cultures significantly decreased the secretion of both IL-17A (top) and IFN-γ (bottom) in obese adults and in lean eldely, but not in lean individuals, further confirming that the effects of aging and obesity are comparable in inducing B cells capable to polarize T cells towards a pro-inflammatory phenotype. The effect of weight loss was evident on the amount of cytokines released in cultures of both total PBMC and B cell-depleted PBMC. Current experiments in our laboratory are investigating the signaling molecules involved in the polarizing effect of B cells. We have preliminary evidence that molecules regulating the metabolic status of B and T cells are involved, in particular ICOS (inducible T cell co-stimulator) expressed on T cells and its ligand, ICOSL, expressed on B cells. Upon encountering ICOSL + B cells, T cells increase glucose uptake and utilization, and differentiate into pro-inflammatory T cells that are glycolytic and pathogenic. This effect requires a cell:cell contact. These findings may be relevant in clinical management not only of obesity but also of other inflammatory conditions and/or diseases, an example is the regulation of inflammation in the joints of patients with rheumatoid arthritis.

## Conclusions

This is a small observational study in which we demonstrate that obesity-induced dysfunctional B cell responses are similar to those induced by aging, and weight loss shows a trend of improvement which is significant in some but not in all obese individuals; however, the effects of body weight loss on mechanistic pathways are largely missing and deserve further investigation. One suggested mechanism that our results have shown is that weight loss reduces inflammaging and intrinsic B cell inflammation, leading to better B cell function. The lack of effects of weight loss in some patients may be due to the fact that these patients retain the strong immunophenotypic imprinting and changes associated with prior obesity, as studies in mice have clearly shown [46, 47]. Although the limitation of including a small number of participants, we believe our results have a significant impact showing that obese individuals undergoing weight reduction may have a higher probability to improve humoral immunity and respond better to infections and vaccination.

## Methods

### Study participants

Experiments were performed using blood isolated from an established cohort of female adult (*n* = 9, 45–55 years, lean with a BMI < 24.9) and elderly (*n* = 9, > 65 years, lean with a BMI < 24.9) individuals, as well as from the blood of female adults with obesity (*n* = 9, 45–55 years, obese with a BMI ≥ 30), before and after a voluntary weight reduction program in which their BMI was reduced 10–34% in 12 months (2 out of 9 individuals remained obese whereas all the other fell in the overweight group). In the voluntary weight loss program, individuals followed the diet aligned with the Diabetes Prevention Program lifestyle intervention behavioral weight loss program, https://dppos.bsc.gwu.edu, and were followed by the doctors and nurses of the Department of Family Medicine at the University of Miami Miller School of Medicine. Diet consisted of < 7% of daily calories from saturated fat; < 200 mg of daily cholesterol; 25–35% of daily calories from total fat, including saturated fat calories; dietary options for increased LDL lowering, including 2 g/day of plant stanols or sterols; 10–25 g/day of soluble fiber; and only enough calories to reach or maintain a healthy weight. In addition, participants will get at least 30 min of a moderate intensity physical activity, such as brisk walking, on most, and preferably all, days of the week. Typically, the goal was weight loss of 7% over 6 months.

All individuals were recruited at the University of Miami Miller School of Medicine. Participants were healthy and were not using medications affecting the immune system. Subjects with type-2 diabetes mellitus, autoimmune diseases, congestive heart failure, cardiovascular disease, chronic renal failure, malignancies, renal or hepatic diseases, infectious disease, trauma or surgery, pregnancy, or under substance and/or alcohol abuse were excluded from the study. All participants signed an informed consent. The study was reviewed and approved by our Institutional Review Board (IRB, protocols #20070481 and #20160542), which reviews all human research conducted under the auspices of the University of Miami.

### Influenza vaccination

All individuals were vaccinated with the Trivalent Inactivated Influenza Vaccine during the during the 2012–2013 and 2013–2014 Influenza vaccine seasons. The composition of the vaccines in the two seasons were: 2012–2013 (A/California/7/2009 (H1N1), A/Victoria/361/2011 (H3N2), B/Wisconsin/1/2010-like (Yamagata lineage)) and 2013–2014 (A/California/7/2009 (H1N1), A/Victoria/361/2011, B/Massachusetts/2/2012). All individuals were vaccinated each year since the 2009–2010 vaccine season. Blood samples were collected before and 4 weeks after vaccination.

### HemAgglutination Inhibition (HAI) assay

The HAI assay was used to measure the serum response to the influenza vaccine. The assay is based on the ability of certain viruses or viral components to hemagglutinate the red blood cells of specific animal species. Antibodies specific for influenza antigens can inhibit this agglutination. Paired pre- and post-immunization serum samples from the same individual were tested simultaneously to evaluate antibody production to the vaccine (and therefore to all viral strains present in the vaccine). Serum inhibiting titers of 1/40 or greater are the defined positive measure of seroprotection against infection.

### PBMC collection

Blood was drawn in Vacutainer CPT tubes (BD 362761), then PBMC were isolated and cryopreserved. PBMC were thawed and cultured at the concentration of 1 × 10^6^/mL in complete medium (c-RPMI) [RPMI 1640 (Gibco ThermoFisher 11,875–093), supplemented with 10% FBS (Gibco 10,437–028), 100 U/mL Penicillin–Streptomycin (Gibco 15,140–122), and 2 mM L-glutamine Gibco 25,030–081)]. FBS was certified to be endotoxin-free. After thawing, viability of the PBMC was checked by trypan blue counting and samples were discarded if viability was < 75%.

PBMCs were stimulated with plate-bound anti-CD3 (eBioscience 16–0037-85) (1 μg/mL) for 48 h, in the presence or absence of B cells, which were removed by magnetic sorting using CD19 microbeads as indicated below (< 0.1% B cells in PBMC after selection). Culture supernatants were collected for the quantification of T cell cytokines by ELISA.

### B cell isolation and in vitro stimulation

B cells were isolated from thawed PBMC by magnetic sorting using CD19 Microbeads (Miltenyi Biotec 130–121-301) according to the MiniMACS protocol (20 µL Microbeads + 80 µL PBS, for 10^7^ cells). Cell preparations were typically > 95% pure.

B cells at the concentration of 10^6^/mL of c-RPMI were stimulated 1–5 days with 1 µg/10^6^ cells of CpG (ODN 2006 In Vivogen). Cells were harvested and mRNA extracted with µMACS mRNA isolation kit (Miltenyi Biotec) to evaluate by qPCR mRNA expression of the transcription factors E47 and T-bet (at day 1). Culture supernatants were collected at day 2 of stimulation to measure the secretion of pro- and anti-inflammmatory cytokines by ELISA.

B cells were also left unstimulated, and within 15 min of magnetic sorting, they were resuspended in TRIzol (Ambion) for RNA extraction to evaluate by qPCR RNA expression of markers of the SASP.

### Enzyme-linked immunoabsorbent assay (ELISA)

Serum IL-6, CRP, TNF-α and leptin were measured by ThermoFisher Scientific kits KHC0062, KHA0031, KHC3013 and BMS2039INST, respectively. Serum MDA-specific IgG antibodies were measured by the MyBioSource MBS390120 kit, whereas serum anti-dsDNA IgG antibodies were measured by the Signosis EA-5002 kit. IL-6, IL-10, IL-17, IFN-γ in culture supernatants were measured by ThermoFisher Scientific kits KHC0062, KHC0104, KAC1591, KHC4021, respectively.

### Flow cytometry

After thawing, PBMC (2 × 10^6^/mL) were stained for 20 min at room temperature with the following antibodies: anti-CD45 (Biolegend 368,540), anti-CD19 (BD 555415), anti-CD27 (BD 555441) and anti-IgD (BD 555778) to measure naive (IgD + CD27-), IgM memory (IgD + CD27 +), switched memory (IgD-CD27 +), and DN (IgD-CD27-) B cells. Up to 10^4^ events in the B cell gate were acquired on an LSR-Fortessa (BD) and analyzed using FlowJo 10.5.3 software. Single color controls were included in every experiment for compensation. Isotype controls were also used in every experiment to set up the gates.

### mRNA extraction and quantitative (q)PCR

To evaluate the mRNA expression of the transcription factors for class switch, the mRNA was extracted from CpG-stimulated B cells at day 1 (to evaluate E47 and T-bet), using µMACS mRNA isolation kit (Miltenyi 130–075-201) following manufacturer’s instructions. The mRNA was eluted into 75 µL of pre-heated (65 °C) elution buffer, and stored at -80 °C until use. To evaluate the expression of SASP markers, total RNA was isolated using TRIzol, according to the manufacturer’s protocol, and stored at -80 °C until use.

Reverse Transcriptase (RT) reactions were performed in a Mastercycler Eppendorf Thermocycler to obtain cDNA. Briefly, 10 µl of mRNA or 2 µl of total RNA at the concentration of 0.5 µg/µl were used as template for cDNA synthesis in the RT reaction. For miRs quantification, RNA was reverse transcribed in the presence of specific primers (provided together with the qPCR primers). In both cases, conditions were: 40 min at 42 °C and 5 min at 65 °C.qPCR reactions using the TaqMan method were conducted in MicroAmp 96-well plates and run in the ABI 7500 machine. Calculations were made with ABI software. Briefly, we determined the cycle number at which transcripts reached a significant threshold (Ct) for each target gene, and for GAPDH or U6 as controls. The difference in Cts between the housekeeping genes (GAPDH or U6) and the target genes was calculated as ΔCt. Then the relative amount of the target gene was expressed as 2^−ΔCt^ and indicated as qPCR values. Reagents and Taqman primers (ThermoFisher), were the following: GAPDH, Hs99999905_m1; E47, Hs01012685_m1; tbx21 (T-bet), Hs00894391_m1; TNF, Hs01113624_g1; IL-6, Hs00985639_m1; TLR2, Hs02621280_s1; TLR4, 00152939_m1; p16^INK4^ (CDKN2A), Hs00923894_m1; p21^CIP1/WAF1^, Hs00355782_m1; U6, 001973; miR-155, 002623; miR-16, 000391; miR-181a, 000480.

### Statistical analyses

To examine differences between 4 groups, two-way ANOVA was used. Group-wise differences were analyzed afterwards with Bonferroni’s multiple comparisons test, with *p* < 0.05 set as criterion for significance. To examine differences between 2 groups, paired Student’s t test (two-tailed) was used. To examine relationships between variables, bivariate Pearson’s correlation analyses were performed. Principal Component Analyses (PCA) were generated using GraphPad Prism version 9 software, which was also used to construct all graphs.

## Data Availability

Data and materials are available upon request to the corresponding author.

## Abbreviations

BMI: Body Mass Index
dsDNA: Double strand DNA
MDA: Malondialdehyde
PCA: Principal Component Analysis
SASP: Senescence-associated secretory phenotype
